# Pharmacological Analyses of Protein Kinases Regulating Egg Maturation in Marine Nemertean Worms: A Review and Comparison with Mammalian Eggs

**DOI:** 10.3390/md8082417

**Published:** 2010-08-24

**Authors:** Stephen A. Stricker, Jose R. Escalona, Samuel Abernathy, Alicia Marquardt

**Affiliations:** Department of Biology, University of New Mexico, MSC03 2020, Albuquerque, NM 87131, USA; E-Mails: escalonarj@gmail.com (J.R.E.); sabernat@unm.edu (S.A.); amarq7@unm.edu (A.M.)

**Keywords:** GVBD, AMPK, MPF

## Abstract

For development to proceed normally, animal eggs must undergo a maturation process that ultimately depends on phosphorylations of key regulatory proteins. To analyze the kinases that mediate these phosphorylations, eggs of marine nemertean worms have been treated with pharmacological modulators of intracellular signaling pathways and subsequently probed with immunoblots employing phospho-specific antibodies. This article both reviews such analyses and compares them with those conducted on mammals, while focusing on how egg maturation in nemerteans is affected by signaling pathways involving cAMP, mitogen-activated protein kinases, Src-family kinases, protein kinase C isotypes, AMP-activated kinase, and the Cdc2 kinase of maturation-promoting factor.

## 1. Introduction

**Figure 1:**
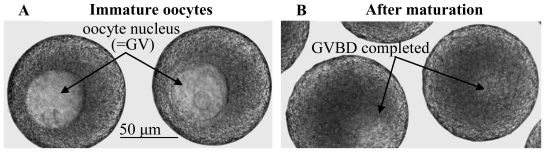
Disassembly of the oocyte nucleus (=germinal vesicle breakdown, GVBD) at the onset of egg maturation. Immature oocytes from a marine nemertean worm possess an intact nucleus (=GV). After maturation is induced, each oocyte undergoes GVBD to become a mature egg.

Animal eggs begin their development in ovaries as immature oocytes, with each oocyte possessing a large nucleus that is referred to as the “germinal vesicle”, or GV (Figure 1). In response to appropriate stimulation, immature oocytes undergo a complex maturation process that starts with the disassembly of the oocyte nucleus during a sequence called germinal vesicle breakdown (GVBD). Following such GVBD and subsequent stages of maturation, the oocyte becomes a mature egg that is capable of developing further once fertilization has been achieved. In most animals, GVBD precedes fertilization, whereas in a few cases, GVBD begins after fertilization has occurred [1]. However, regardless of the timing of GVBD relative to fertilization, all eggs must eventually mature in order for subsequent development to proceed normally. Thus, as might be expected for such an essential step in animal development, the underlying mechanisms of egg maturation have been intensively analyzed.

As a result of such analyses, egg maturation has been shown to involve various regulatory proteins, whose activities are often modulated by site-specific phosphorylations. Such phosphorylations in turn depend on upstream kinases that add phosphate groups to their protein targets and associated phosphatases that cause dephosphorylation, thereby providing a counterbalanced mechanism for modulating protein activity. In order to monitor these phosphorylations, early studies often utilized *in vitro* assays of individual kinases and targets that were purified from cells and incubated with radioactive phosphate to measure phosphate incorporations on a case-by-case basis. However, the advent of phospho-specific antibodies and their usage in conjunction with immunoblotting techniques have now provided an alternative method for assaying multiple phosphoproteins in total cell lysates.

**Figure 2:**
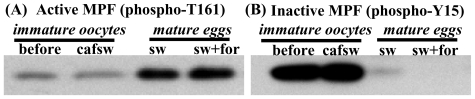
Differing phosphorylation status affecting MPF activity and egg maturation. Blots showing phosphorylation status of the ~32 kD Cdc2 kinase of MPF in oocytes of a marine nemertean worm: (**A**) Active MPF, which has phosphorylated T161 and non-phosphorylated Y15 on Cdc2, is at low levels in immature oocytes [before treatment (before) or after 2 hr in calcium-free seawater (cafsw)] *vs.* at high levels in mature eggs [after 2 hr in seawater (SW) or SW + 10 μM of the cAMP elevator forskolin (for)]; (**B**) Inactive MPF (high p-Y15; low p-T161) is high in immature oocytes and low in mature eggs (see Section 2.6 for more details).

A major impetus for applying such methods to studies of egg maturation can be traced back to 1971, when it was shown that the cytoplasm of maturing oocytes contains a “maturation-promoting factor” (MPF) that stimulates GVBD after being injected into immature oocytes [2]. Subsequent analyses have revealed that MPF is a heterodimer consisting of a regulatory protein called cyclin B plus a kinase component referred to as Cdc2. Moreover, such investigations have demonstrated that MPF’s activity can be either inhibited or stimulated by different types of phosphorylations (Figure 2)[3–7].

Since the discovery of MPF, cellular signaling pathways that ultimately regulate the phosphorylation of MPF have been investigated in various animals, particularly within mammals, where results obtained from such studies can have important veterinary and clinical applications. For example, mice represent an intensively analyzed mammalian model, while considerable work has also been conducted on egg maturation in other rodents such as rats, as well as in primates and domesticated mammals, including cows, pigs, and horses. However, mammalian eggs are not optimally suited for all analyses. For example, compared to most other animals, relatively low numbers of eggs can be obtained at any one time from a fertile mammal. Moreover, given that intraovarian oocytes of mammals develop within complex follicles that are subject to input from multiple sources, it can be difficult to fully replicate the *in vivo* cues that mediate egg maturation in mammals. Similarly, the external layers of residual follicle cells (=“cumulus cells”) that surround mammalian oocytes after they have been ovulated from the ovary can in some cases complicate interpretation of experimental results.

As an alternative to analyzing egg maturation in mammals, various non-mammalian animals, including marine invertebrates, have also been investigated. For example, egg maturation has been studied in marine worms belonging to the phylum Nemertea [8]. Such nemerteans (or “ribbon worms”) typically have separate sexes, and in the case of a ripe female, numerous ovaries are present along the length of the body. During the breeding season that usually occurs in spring or summer, most nemerteans discharge their gametes directly into the sea [9]. Moreover, unlike in mammals, nemerteans characteristically lack follicle cells during intraovarian stages of egg development and around their post-spawned eggs [10]. Thus, small pieces of gravid nemerteans are capable of yielding hundreds to thousands of follicle-free oocytes that start GVBD ~15–30 min after treatment with seawater (SW). Conversely, nemertean oocytes can be kept immature in calcium-free seawater (CaFSW) before being immersed in SW to overcome the inhibitory effects of CaFSW [11]. Moreover, not only is GVBD stimulated by replacing CaFSW with SW, but agents that elevate intraoocytic levels of cyclic 3′,5′-adenosine monophosphate (cAMP) also cause maturation when added directly to CaFSW.

**Figure 3:**
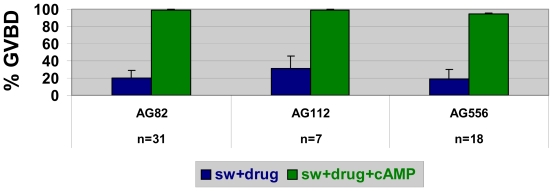
Differing modes of seawater- *vs.* cAMP-induced egg maturation in nemerteans. When added to seawater (SW), various drugs (e.g., tyrphostin inhibitors of tyrosine kinases [AG82, AG112, AG556 at 50–100 μM]) significantly reduce egg maturation (=blue bars) compared to controls in SW alone (control GVBD in SW=95.9 ± 7%; N = 56), whereas co-treatments of tyrphostins plus cAMP elevators (=green bars) in SW restore GVBD, indicating SW and cAMP elevators utilize different maturation-inducing pathways.

Exactly why nemertean GVBD is triggered by SW and blocked by CaFSW has not been fully elucidated, but supplementing artificial seawaters with Ca^2+^ only partially restores GVBD, indicating that natural SW contains additional GVBD-inducing substance(s) other than just Ca^2+^ itself [11]. Moreover, although it is possible that SW-stimulated oocytes also undergo some sort of rise in cAMP during GVBD, the precise patterns of these potential cAMP elevations and/or their mediating pathways must differ following treatment with SW *vs.* cAMP elevators. Such a conclusion is based on the fact that various kinds of drugs block SW-induced GVBD, whereas simply combining these drugs in SW with cAMP elevators fully restores GVBD, thereby demonstrating that non-identical GVBD-inducing mechanisms are triggered by SW *vs.* cAMP elevators (Figure 3). Accordingly, the multiple modes of achieving GVBD indicated by such cAMP-induced rescues provide a means for assessing whether the blockages of GVBD observed in SW solutions of pharmacological agents are just due to oocyte morbidity or to a drug effect that can be reversed by alternative signals mediated by cAMP elevators.

As opposed to numerous relatively recent synopses that are available for mammalian egg maturation [12–15], this review summarizes for the first time previous studies on SW- *vs.* cAMP-induced maturation in nemertean eggs. In particular, the roles played by protein phosphorylations in maturing nemertean eggs are covered, while focusing on signaling pathways involving cAMP, mitogen-activated protein kinases (MAPKs), Src-family kinases (SFKs), protein kinase C isotypes (PKCs), AMP-activated kinase (AMPK), and MPF. In addition, studies of nemertean egg maturation are also briefly compared and contrasted with results previously reported for mammals in order to synthesize common and divergent patterns of egg maturation in these two distantly related groups of animals. In so doing, such comparisons relate to an overall goal of this special issue that is aimed at elucidating the functional significance of proteins expressed by various marine organisms.

## 2. Results and Discussion

### 2.1. Elevations in intraoocytic cAMP trigger GVBD in nemerteans as opposed to generally inhibiting GVBD in mammals

In nemerteans, pre-GVBD increases in cAMP have been observed by confocal microscopy in maturing oocytes that had been microinjected with a vital probe for tracking cAMP, and based on several lines of evidence, such intraoocytic cAMP elevations serve to trigger egg maturation [16]. For example, GVBD in nemerteans can be elicited by a variety of cAMP-elevating drugs with diverse modes of action, including: (1) membrane-permeable cAMP; (2) forskolin, which stimulates the cAMP-synthesizing activities of adenylate cyclases (ACs); (3) such phosphodiesterase (PDE) inhibitors as IBMX and Ro-20-1724 that block the degradation of cAMP; and (4) serotonin (“5-HT”), which may act as both an AC stimulator and a PDE inhibitor [16]. In addition, inhibitors of the main downstream target of cAMP, protein kinase A (PKA), block GVBD in nemerteans [16], collectively indicating that cAMP signaling stimulates maturation in nemertean eggs.

Conversely, based on various studies, GVBD in mammals tends to be blocked by elevations in intraoocytic cAMP, and as opposed to its antagonistic effect on GVBD in nemerteans, inhibiting the cAMP target PKA can trigger mammalian egg maturation [17–23]. Such increased levels of cAMP in immature mammalian oocytes are mediated via signals received from surrounding follicle cells [24–27]. Accordingly, mammalian oocytes that are removed from their follicles can undergo a decline in cAMP followed by the onset of GVBD. Thus, to prevent denuded oocytes from initiating such “spontaneous maturation”, mammalian oocytes are typically incubated continually in various cAMP-elevating drugs to maintain elevated cAMP levels and an inhibition of GVBD for prolonged periods. However, a variation on the inhibitory effects of cAMP has been documented with data showing that a short elevation in intraoocytic cAMP actually leads to the stimulation, rather than inhibition, of GVBD in mice, as such a cAMP pulse causes a GVBD-stimulating increase in AMPK activity, which normally does not occur in immature oocytes subjected to prolonged elevations in cAMP [28].

In any case, although the exact reasons why intraoocytic cAMP elevations tend to generate opposite results in mammalian *vs.* nemertean eggs have not been determined, it is clear that nemerteans are not the only animals to differ from mammals, as several other marine invertebrates are also known to undergo cAMP-induced GVBD [29–33]. Accordingly, compared to mammals, nemertean eggs may react differently to cAMP owing to alternative kinds of upstream modulators and downstream targets of cAMP (e.g., PDEs, PKAs, AMPKs) and/or to differing subcellular localizations of key maturation regulators [34]. Moreover, such differences in turn may somehow relate to the presence of follicle cells in mammals and their absence in nemerteans, given that in nemerteans and the other marine invertebrates where stimulation of GVBD by cAMP has been reported to occur, follicle cells are either lacking or apparently not as well connected to immature oocytes as in mammals [35,36].

### 2.2. ERK 1/2 MAPKs are activated before, but not required for, GVBD in nemerteans

**Figure 4:**
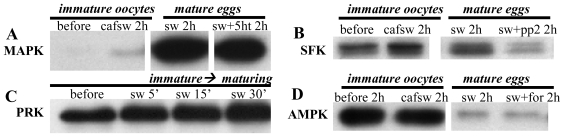
Protein phosphorylations in immature oocytes *vs.* mature eggs of nemerteans. Blots probed with phospho-specific antibodies to track active forms of: (A) ERK 1/2 mitogen-activated protein kinase (MAPK), (B) Src family kinases (SFK); (C), Protein kinase C related kinase (PRK), or (D) AMP-activated kinase (AMPK). 5ht = serotonin; cafsw = calcium-free seawater; for = forskolin; pp2 = SFK inhibitor; sw = seawater.

Mitogen-activated protein kinases (MAPKs) constitute a diverse family of Ser/Thr kinases that can play key roles in regulating cell division [37–40]. For various eggs, MAPKs of the type called ERK 1/2 (extracellular signal regulated kinases 1/2, =“MAPKs 3/1”, or simply MAPK hereafter) represent the most intensively analyzed forms of these kinases. Accordingly, in nemerteans, MAPK activity has been shown to be low in immature oocytes, based on immunoblots using a widely employed phospho-specific antibody that recognizes the dually phosphorylated and thereby activated form of MAPK (Figure 4A)[41–44]. Conversely, in response to stimulation by either SW or cAMP elevators, MAPK becomes activated prior to GVBD and typically remains elevated post-GVBD as such mature eggs become arrested at metaphase I of meiosis [41–44]. Similarly, a maturation-induced increase in MAPK activity has also been verified in kinase assays using a known target of active MAPK [41].

To analyze potential upstream regulators of MAPK activation during nemertean egg maturation, immature oocytes have been incubated in SW solutions containing inactivators of Raf-1, a MAPK kinase kinase (MAPKKK). Such treatments specifically block SW-induced MAPK activation and GVBD [43]. Conversely, adding cAMP elevators restores both MAPK activity and GVBD [43]. Similarly, several kinds of tyrphostin-type inhibitors of tyrosine kinases also block MAPK activation and GVBD in response to SW, whereas both of these processes are re-established by co-treatment with cAMP elevators [35]. Collectively, these findings suggest that MAPK activation and GVBD during SW stimulation depend on Raf-1 activity and some as-of-yet undetermined tyrosine kinase. Alternatively, cAMP signaling can apparently activate MAPK and trigger GVBD via a Raf-1-independent mechanism that does not involve the activity of tyrosine kinases or other substrates targeted by the tested tyrphostins.

Moreover, these results suggest that nemertean egg maturation is normally associated with an increase in MAPK activity. However, to determine if in fact MAPK is actually required for GVBD, immature oocytes have been incubated in several inhibitors of MAPK kinase (MAPKK) prior to stimulation by SW or cAMP elevators. In response to the MAPKK inhibitors PD98059 and CI1040, MAPK activation remains downregulated during either SW or cAMP stimulation, and yet such specimens consistently undergo GVBD [44]. Similarly, the MAPKK antagonist U0126 also fails to block cAMP-induced GVBD while inhibiting MAPK activity, and some U0126-treated oocytes still continue to mature in SW. However, in other oocyte batches, SW-induced GVBD is indeed lower in the presence of U0126 [42–44]. Nevertheless, other drugs that inactivate MAPK (e.g., the Src inhibitors PP2 and SU6656: see next section) also allow both SW- and cAMP-induced GVBD in the absence of marked MAPK activation [43]. Such findings demonstrate that although tests utilizing Raf-1 and tyrosine kinase inhibitors suggest MAPK is required for SW-induced GVBD, the fact that oocytes still undergo GVBD without displaying marked MAPK activity when treated with MAPKK or Src antagonists reveals that maturation can occur via a MAPK-independent mechanism. Accordingly, the blockage of SW-induced GVBD by Raf-1, tyrosine kinase inhibitors, and U0126 is apparently due to one or more drug effects other than just MAPK inhibition.

Aside from analyses of GVBD prior to insemination, additional investigations have revealed that during fertilizations in the presence of MAPK inhibitors, polar bodies are still formed as eggs continue with the first cell cycle [44]. Thus, as with GVBD, at least some post-GVBD processes that occur during egg maturation in nemerteans can proceed without pronounced MAPK activation.

As for mammals, the potential roles played by intraoocytic MAPK activation in maturing eggs appear a bit more varied, as there is evidence indicating that an increase in MAPK occurs within oocytes of some species prior to GVBD and may be required for initiating maturation [14,45,46]. Conversely, in other mammals, MAPK does not become activated in oocytes until after GVBD is elicited and is thus not required for GVBD [14,45,46]. Nevertheless, in spite of these differences, MAPK must be activated in follicle cells surrounding mammalian oocytes in order for egg maturation to proceed properly [47,48]. Moreover, as reviewed elsewhere [14,45,46], intraoocytic activation of MAPK can certainly play important roles during post-GVBD stages of mammalian egg maturation, including the proper formation of meiotic spindles and polar bodies.

### 2.3. Src-family kinase activation does not appear to be needed for GVBD in nemerteans

Src-family kinases (SFKs) are ~52–62 kD non-receptor tyrosine kinases that include such examples as Src, which is the first SFK member documented to upregulate egg maturation [49]. To determine if SFKs stimulate GVBD in nemertean oocytes, immature specimens have been incubated in SW solutions of the SFK inhibitors PP2 and SU6656. Such treatments fail to block GVBD, even though the drugs are effective in deactivating MAPK, and at least in the case of PP2, can reduce SFK activation as judged by blots probed with a phospho-specfic antibody to active SFKs (Figure 4B)[50]. Moreover, not only does SFK activity fail to exhibit a marked increase in mature eggs *vs.* immature oocytes, treating CaFSW-incubated immature oocytes with SFK inhibitors actually causes a slight increase in GVBD over baseline levels in CaFSW alone (unpubl. obsv.). Collectively, such findings suggest that SFKs are not required for nemertean GVBD, and in fact, can serve to inhibit it.

Similarly, in one study of mouse oocytes, inactivation of SFK by the pharmacological inhibitor SKI606 also fails to prevent GVBD and actually accelerates the onset of maturation [51]. Alternatively, another investigation reports a blockage of GVBD following the use of PP2 to downregulate SFK activity in mouse oocytes [52]. Although the reasons for this discrepancy have not been determined, such variable effects may be due to differing specificities of action for the two SFK inhibitors and/or to variations in the strains of mice and the formulations of culture media used in these experiments [51]. In any case, data obtained for the roles played by SFKs during subsequent stages of mammalian egg maturation consistently demonstrate that SFK activity is required for post-GVBD progression to Metaphase II (Met II) arrest as well as for proper resumption of meiosis following Met II arrest [51,53–56].

### 2.4. Inhibitors of atypical PKCs and PKC-related kinases (PRKs) block SW-, but not cAMP-induced, GVBD in nemerteans

Eukaryotic cells can express 11 isotypes of protein kinase C (PKC) that are usually assigned to three classes—conventional, novel, and atypical [57–59]. To attain full activity, conventional PKCs (α,βI,βII,γ) require calcium ions (Ca^2+^) and diacylglycerol (DAG)[or a DAG analog such as 12-*O*-tetradecanoylphorbol 13-acetate (TPA)]. Novel isotypes (δ,ɛ,μ,η,θ), on the other hand, are activated by DAG but not Ca^2+^, and atypical PKCs (ζ, λ/ι) are insensitive to both Ca^2+^ and the type of DAG that stimulates conventional/novel isotypes [57–59]. In spite of such differences, PKCs share amino acid sequences that are typically phosphorylated in a conserved fashion during kinase activation and can thus be monitored via phospho-specific antibodies as a means of assessing PKC activity [36].

Based on such antibodies, nemertean oocytes activate one or more PKCs during maturation induced by SW or cAMP-elevating drugs [36]. Accordingly, in SW-stimulated oocytes, GVBD is inhibited by broadly acting PKC antagonists such as bisindoylmaleimide (BIM)-I or BIM-IX, whereas co-treatment with SW solutions of BIM-I or BIM-IX plus a cAMP elevator restores GVBD, suggesting that SW-, but not cAMP-induced GVBD depends on PKC activation [36]. In tests to determine which specific PKC regulates GVBD, immunoblots fail to provide evidence for TPA-sensitive conventional or novel PKCs [36]. Moreover, inhibitors of such PKCs do not prevent SW-induced GVBD, and instead of stimulating GVBD, as would be expected if TPA-sensitive PKCs triggered maturation, TPA itself actually downregulates GVBD [36]. Alternatively, maturing oocytes possess phosphorylated forms of TPA-insensitive atypical isotypes as well as a PKC-related kinase (PRK), which would also presumably be insensitive to TPA (Figure 4C)[36]. Accordingly, inhibitors of atypical PKC/PRK signaling block SW- but not cAMP-induced GVBD [36], collectively suggesting that SW-, but not cAMP-induced, GVBD requires activation of an atypical PKC and/or PRK and that conventional/novel PKC activity may inhibit GVBD.

After GVBD is completed in nemerteans, a positive feedback loop is apparently established between PKC and MAPK signaling pathways such that MAPK and one or more unidentified ~80–95 kD conventional/novel PKCs stimulate each other and thereby keep the activities of both kinds of kinases elevated within metaphase-I-arrested mature eggs [44]. Accordingly, following fertilization of these eggs, such positive feedback is uncoupled, as both kinase types normally decrease their activities [44]. Moreover, for fertilizations conducted in the presence of BIM-I to inhibit PKCs, sperm incorporation and polar body formation still occur, but first cleavage is blocked, suggesting that although certain aspects of post-fertilization maturation can continue in the absence of PKC activation, full progression through the first cell cycle requires the activity of one or more as-of-yet unidentified PKC isotypes [44].

As for mammals, whether PKC up- or downregulates egg maturation appears to vary depending on the species examined and/or the experimental design used [60]. In mice, at least some of this variability could be due to the fact that PKC activation in surrounding follicle cells typically triggers GVBD, whereas stimulating PKC within immature oocytes tends to inhibit GVBD [60]. Moreover, intraoocytic PKC activity may not always have the same effect on GVBD in mice, as some PKC isotypes can either down- or upregulate GVBD depending on the timing of their activation and their localization relative to the nuclear *vs.* cytoplasmic compartments of the oocyte [61]. In any case, as opposed to the case in nemerteans where SW-induced GVBD apparently requires the activity of atypical PKC and/or PRK, most studies using pharmacological modulators of PKC indicate that GVBD in rodent eggs is inhibited by intraoocytic PKC activations that in turn appear to involve conventional isotypes [60–66]. Similarly, the continued production of polar bodies following fertilization of BIM-I-treated nemertean oocytes contrasts with the blockage of polar body formation in similarly treated mammalian oocytes that has previously been reported in many [67–69] but not all [70] analyses of PKC signaling. In any case, in order to clarify further the functions of PKC isotypes during egg maturation, pharmacological modulators of PKC signaling need to be used judiciously owing to potential problems associated with such drugs, and wherever possible data obtained with these modulators should be supplemented with results using genetic knockouts and RNA interference strategies [59].

### 2.5. Stimulators of AMP-activated kinase block SW- but not cAMP-induced GVBD in nemerteans

In eukaryotes, decreased nutrient availability and various other stresses can increase intracellular AMP:ATP ratios and thereby stimulate AMP-activated kinase (AMPK), a highly conserved regulator of energy states and cell cycle progression [71–73]. To test the possible roles played by AMPK activation during egg maturation in nemerteans, oocytes have been frozen at increasing times following stimulation with either SW or cAMP elevators before being subjected to immunoblotting with phospho-specific antibodies to AMPK and its downstream target acetyl CoA carboxylase [74]. Such studies reveal that AMPK activity is normally high in immature oocytes and subsequently reduced during either SW- or cAMP-induced GVBD (Figure 4D). Accordingly, adding various AMPK agonists to SW maintains AMPK activity and blocks SW-induced GVBD [74]. Conversely, co-treating oocytes with SW solutions of such AMPK agonists plus cAMP elevators restores AMPK deactivation and allows GVBD to occur [74]. Collectively, these findings indicate that active AMPK normally blocks nemertean GVBD and that cAMP elevators can downregulate AMPK via a mechanism that is independent of the AMPK-deactivating pathway(s) elicited by SW.

In mice, AMPK activation triggers GVBD in either cumulus-enclosed or denuded oocytes [75–77]. Conversely, studies of cows and pigs have shown that activating AMPK in cumulus-enclosed oocytes can block GVBD [78–80]. Thus, the effects of AMPK on mammalian egg maturation may vary, depending on the species analyzed, although, at least in cows, further analyses are needed to determine if such inhibitory effects of AMPK activation are due to direct responses within the oocyte itself or to AMPK-mediated signals transmitted from surrounding follicle cells to the oocyte [81].

### 2.6. Egg maturation in nemerteans requires activation of pre-MPF following stimulation by either SW or cAMP elevators

Although there are a few exceptions where GVBD can be triggered by RINGO/SPDY-mediated stimulation of Cdc2 or perhaps by other regulators [82,83], the initiation of GVBD in animals generally requires activation of MPF itself. In order to activate MPF in oocytes of the frog *Xenopus* and many other animals, the Cdc2 kinase of inactive “pre-MPF” (=cyclin B bound to inactive Cdc2) needs to become properly phosphorylated (Figure 2). Thus, as pre-MPF becomes activated, the T161 residue of Cdc2 remains phosphorylated by cyclin-dependent kinase activating kinase (CAK), while an inhibitory phosphate placed on Y15 of Cdc2 by Myt-1/Wee-1 kinases is ultimately removed by one or more isotypes of a phosphatase called Cdc25 [4]. Alternatively, in some fish and non-*Xenopus* amphibians, MPF activation involves the *de novo* synthesis of cyclin B and its binding to a form of Cdc2 that is properly phosphorylated at just T161 [7,84].

To determine if nemertean GVBD depends on a dual-phosphorylation mode of activating pre-MPF, immature oocytes have been incubated with various MPF inhibitors and subsequently stimulated with either SW or cAMP elevators. In controls lacking the MPF inhibitors, maturing eggs show a rise in T161 phosphorylation and a decrease in Y15 phosphorylation (Figure 2)[43]. Similarly, MPF inhibitors serve to deactivate MPF by reducing the stimulatory T161 phosphorylation and by maintaining relatively high levels of the inhibitory Y15 phosphorylation. Moreover, during both SW and cAMP stimulation, GVBD is blocked by MPF inhibitors. However, compared to SW-incubated oocytess, higher doses of MPF inhibitors are needed for preventing cAMP-induced GVBD, and such drug treatments usually allow a greater amount of MAPK activation in the presence of cAMP elevators [43], collectively suggesting that although both modes of inducing maturation require MPF activity, alternative pathways for activating pre-MPF may be utilized in SW- *vs.* cAMP-induced GVBD.

Accordingly, given that inhibitors of protein tyrosine phosphatases (PTPs) can inhibit SW- but not cAMP-induced GVBD [43], more specific blockers of the dual-specificity phosphatase Cdc25 have been tested and also found to prevent SW-induced GVBD. Conversely, such blockage is routinely rescued by cAMP elevators, suggesting that MPF activation in SW requires Cdc25 activity, whereas cAMP signaling is able to promote MPF activation via an alternative pathway of downregulating Y15 phosphorylation. Conversely, several inhibitors of phosphatases that dephosphorylate Ser-Thr residues, including the phosphatase inhibitor okadaic acid (OA), fail either to block GVBD or to trigger maturation in CaFSW-incubated immature oocytes [43], suggesting that pathways involved in nemertean GVBD may not be fully dependent upon phosphatases affected by these inhibitors.

In mammals, various studies have confirmed that MPF activation is generally required for GVBD. Moreover, such MPF activation can depend on a dual-phosphorylation mode of pre-MPF conversion into active MPF involving maintenance of T161 phosphorylation and depletion of Y15 phosphorylation [4,14]. Accordingly, as with nemerteans, regulation of Cdc25 activity can play a crucial role during MPF activation in mammalian oocytes [85,86]. However, unlike what has been reported for nemerteans where the Ser/Thr phosphatase inhibitor OA fails to block or trigger GVBD, mammalian oocytes can undergo maturation in response to OA [87,88].

## 3. Conclusions

**Figure 5:**
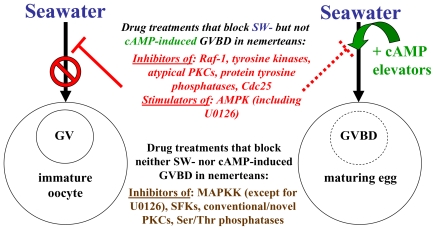
Effects of inhibitors on egg maturation induced by seawater *vs.* cAMP elevators in marine nemertean worms (for further clarification and explanation of abbreviations, see text).

Based on data reviewed above, nemertean eggs are triggered to mature via multiple intracellular pathways that are set in motion by either seawater stimulation or by elevators of cAMP (Figure 5).

In several important ways (e.g., cAMP and AMPK signaling), what triggers GVBD in nemerteans can have an opposite effect on mammalian egg maturation, and *vice versa* (Table 1).

However, although nemertean and mammalian oocytes can utilize similar intraoocytic signaling components to modulate GVBD in opposing fashions, the follicular compartment of mammals also interacts with intraoocytic signals, thereby forming an integrated system in mammals. Accordingly, it is important to compare signaling within the entire mammalian follicle to that in follicle-free nemertean oocytes. When considered in this way, signaling pathways in nemertean oocytes often resemble those in mammalian follicle cells, whereas the intraoocytic pathways of mammals can exhibit opposite effects to either nemertean oocytes or surrounding follicle cells.

In any case, it is clear that further comparative analyses are needed to elucidate both common themes and diverging patterns of egg maturation in nemerteans *vs.* mammals. Such studies should take advantage of the specific benefits of using these types of eggs (e.g., sequenced genomes and available genetic knockouts for mammals *vs.* ready supply of follicle-free nemertean oocytes that because of their multiple modes of triggering maturation provide built-in controls for pharmacological analyses). Collectively, such investigations will hopefully supplement those reviewed here and thereby help to clarify how various phosphoproteins and other regulatory molecules expressed by such marine organisms as nemertean worms can control the same fundamental process of egg maturation that we and other mammals must successfully complete in order to develop properly.

## 4. Experimental

Eggs were typically collected from an undescribed species of the nemertean *Cerebratulus* during summers on San Juan Island, WA, USA. Alternatively, additional analyses were also carried out with eggs obtained from other San Juan Island nemerteans [10] or from *Cerebratulus lacteus* that was purchased from the Animal Supply Department of the Marine Biology Laboratory (Woods Hole, MA) [89]. After removal from ovaries, immature oocytes were routinely pre-incubated in ice-cold calcium-free seawater (CaFSW) for 1.5–2.5 hr to reduce spontaneous GVBD [90,91]. Oocytes were then dejellied via a 150-μm Nitex filter to enhance oocyte pelleting and transferred to SW- or CaFSW-solutions maintained at typical ambient SW temperatures (~12–14 °C) with or without added drugs typically purchased from: Enzo Life Sciences (Plymouth Meeting, PA); LC Labs (Woburn, MA); Sigma (St. Louis, MO); or Tocris (Ellisville, MO). To determine optimal concentrations of drugs to be used, dose-response curves were generated for GVBD, and from these curves, doses occurring between the ED_50_ (effective dose for 50% of the oocytes) and the maximally effective concentrations were routinely adopted in order to achieve a trade-off between increased effectiveness and reduced likelihood of off-target effects [92]. For immunoblotting, total cell lysates were obtained from liquid-nitrogen- frozen oocytes using lysis buffer containing inhibitors of proteases and phosphatases, and after running such lysates on 4%/10% SDS PAGE gels that were cast with BioRad acrylamide (#161-0158), the separated proteins were transferred to PVDF membranes at 100V for 60–75 min and subsequently probed with primary antibodies from Cell Signaling Technology (Danvers, MA) or Millipore (Billerica, MA)[35,41–45,74]. Densitometry of background-subtracted bands was routinely performed on at least three blots from two or more females, and in cases with ambiguous results, statistical analyses were conducted as described previously [41–45].

## Figures and Tables

**Table 1:** Summary of effects of various signals on GVBD in nemertean *vs.* mammalian eggs.

Signal regulating egg maturation:	Positive (+) or negative (−) effect of signal on GVBD	
	in nemerteans [refs] (notes):	in mammals [refs] (notes):
Intraoocytic cAMP elevation	+ [16,35,36,43]	− [17–27]
ERK 1/2 MAPK activation	+ [41–44] (a)	+ [14,45–48] (a,b)
Src family kinase activation	− [50]	− [51] or + [52]
Protein kinase C activation	+ [36,44] [c]	− [60–66] (d)
Intraoocytic AMP kinase activation	− [74]	+ [75–77] (e)
Increased Cdc2 kinase activity via conversion of pre-MPF into MPF	+ [43]	+ [4,14]

(a) Although intraoocytic ERK 1/2 MAPK activation may serve to stimulate GVBD, such activity is generally not required for GVBD to occur.

(b) ERK 1/2 MAPK activation in follicle cells is required for GVBD.

(c) Atypical PKCs and/or PKC-related kinase are required for GVBD; conventional/novel PKCs may inhibit GVBD.

(d) Activation of intraoocytic PKCs tends to inhibit GVBD, although the timing and precise localization of such activation may also cause stimulation of GVBD [61].

(e) Intraoocytic AMPK activation stimulates GVBD in mice; activation of AMPK in follicles of pigs and cows can inhibit GVBD [78–81].
